# Diseases of the Circulatory System

**Published:** 1895-04-06

**Authors:** 


### Progress in Medicine.

### DISEASES OF THE CIRCULATORY SYSTEM.

*(Continued from page 460, Vol. XVII.)*

**Significance of Symptoms.**—Huchard[17] thinks that "brady-diastole" or prolongation of the diastole is a sign of most serious import when occurring in cerebral hæmorrhages, comatose states, and arterial degenerations. The cause of inorganic insufficiency of the mitral valve is referred by Dombrowski[18], not to dilatation of the orifice, but to displacement of the papillary muscles, which prevents closure of the valve. Romberg, however, insists that this is contrary to the known action of those muscles.[19] A new diagnostic sign in anæmia is announced by Verstraeten[20] in the presence of a *bruit de diable* just below the inferior border of the liver, and about three-quarters of a centimetre to the right of the median line. It is produced in the inferior vena cava, and occurs in anæmia, but not in hepatic cirrhosis. The signs of dilatation of the right ventricle are discussed by Stacey Wilson[21]. The chief dilatation in adolescence occurs upwards and to the left, so that the pulmonary valves may be under the first interspace, and the ventricle may extend to the left nipple line in the second space. Some rotation of the heart takes place at the same time, so that the apex often appears in the fourth space external to the nipple line. In later life the dilatation may extend a little to the right of the sternum, as well as upwards. The signs are pulsation in second and third left interspaces and in the veins of the neck, while the apex beat is in the fourth space; increased area of dulness; a pulmonary systolic murmur; accentuated second sound *bruit de diable*, signs of tricuspid regurgitation; and a *bruit de galop*. Several of these signs may be absent.

A discussion on the significance of an accentuated second sound has been carried on by Ringer and Fear, Ewart, and others. The two former show that a high tension causes a loud second sound, but that it also occurs sometimes in the opposite condition, and that various factors affecting the thickness and density of the overlying tissues may influence its production.[22] In short, that there is great difficulty in explaining its production in any given case. Ewart[23] argues that the pulmonary second sound is singularly limited in its area of conduction, while a sound from the aorta is heard far and wide over the cardiac area and beyond it, as shown by the anatomical configuration of the parts. The rare pulsus bisferiens (as well as the anacrotic one) has been usually regarded as produced by aortic stenosis. It is defined as follows: "Both percussion and tidal waves are well represented, but the latter, instead of being rounded and sustained, forms a sharp angle, Moreover, the second wave begins low, and the two waves reach the same level or nearly so. The dicrotic wave follows, and its degree of development varies." Now G. Steell has given reason to think that this pulse may occur without aortic stenosis, but rather in regurgitation. J. Mitchell Clarke describes an undoubted case where imcompetence but no stenosis was present on post-mortem examination. He also reports[24] two other cases in which the pulse occurred without any evidence of stenosis though unconfirmed by a similar examination, and believes that there are two varieties; one (I.) occurs as above in regurgitation, and the other (II.) which is merely a species of the anacrotic pulse with the second wave rounded and beginning high, does actually present itself in stenosis. Even the true anacrotic pulse is shown by Steell[25] not to be always indicative of stenosis, and the P. bisferiens has been observed where there was no incompetence according to a paper of Mahomed's.

The condition of the blood in the cyanosis of congenital heart disease has been analysed by various observers. Banholtzer[26] found in one case that the hœmoglobin stood at 160 per cent. of normal, and the red cells at 9,450,000 in place of 5,000,000. A. Morrison[27] gives two others in which the figures were 110 per cent. and 92 per cent. for hœmoglobin, and the corpuscles 8,470,000 and 6,700,000, and he quotes similar instances from other writers. He believes the increase to be too great to be accounted for by concentration of the blood, but that it is in a sense compensatory. The corpuscles being insufficiently oxygenated, their functions and wear and tear are lessened, and their individual duration is increased, so that their total number becomes much greater than usual. The increase occurs not only in congenital cases, but also in others where cyanosis is present. A very rare case of pulmonary incompetence occurring in adult life and confirmed post-mortem is recorded by T. Oliver.[28] The diastolic murmur was conveyed down the left of the sternum to the apex of the right ventricle, while the pulse was not that of aortic regurgitation. Cyanosis, distended veins in the neck, and profuse epistaxis were also noted. Another case of interest[29] is one of embolic pneumonia, caused by endarteritis of the pulmonary artery, which was associated with congenital stenosis. Shingleton Smith who reports the case, remarks that right endocarditis and its resulting embolic pneumonia are well recognised, while the arteritis was probably localised at this spot by the existing contraction of the vessel.

**The Physics of the Circulation.**—The effect of gravity on the circulation if all the vessels were relaxed, would, of course, render the brain bloodless in the upright position. Hence an exact co-ordination must be maintained between the contractile forces of all the vessels and the pressure due to gravity. In a paper before the Royal Society Leonard Hill showed that this constant compensation is effected by the splanchnic vaso-motor mechanism.[30] When this is damaged by shock, asphyxia, or chloroform the cerebral circulation ceases. Chloroform paralyses this compensatory mechanism more rapidly than ether, hence the danger of elevating the head in chloroform narcosis. Probably, too, emotional syncope is due less to heart failure than to paralysis of the splanchnic nerves. In short, when this protective agency ceases to act, the blood at once accumulates in the veins and the lowest part of the body.

Brunton, in the Harveian oration, carefully discussed the mechanics of the muscular circulation.[31] The blood supply may be principally sent to either the skin, the intestinal tract, or through the muscles, by vaso-motor regulation. Muscular contraction, while it compresses the vessels of that muscle, leads to dilatation of those vessels by stimulation of the vaso-dilator nerves. Thus gentle exercise may lead to general dilatation of the vessels and lowering of blood pressure, since the vessels of the muscles can easily contain all the blood of the body, while violent exercise by compressing the vessels may raise pressure. Again, in the latter case the stimulation of the nerves from the muscles to the heart vastly increases its rate, thus again raising the pressure. Hence the pain produced by sudden exertion in a weak heart, whereas continued and especially gentle exercise by dilating the vessels of the muscles renders circulation more easy. The bearing of this physiology on massage and graduated exercises in heart disease is obvious.

**Raynaud's Disease.**—Morton discusses[32] three cases, the etiology of each of them being different from the others. In one the terminal phalanges would at first turn white, while the rest of the hand was livid. Eventually this became permanent, and gangrene of a phalanx followed. This, he thinks, was pure vaso-motor spasm. In the second there had long been interstitial nephritis. Acute pain in the fingers occurred, with lividity, and finally gangrene. The arterial fibrosis was probably the agent here. The third patient had syphilis some years previously, the fingers were white and the hands livid, while the nails were more or less degenerated. The syphilitic cause was shown by the improvement under specific remedies. Urquhart[33] describes two cases in insane persons where the feet were attacked, where neither neuritis or vascular occlusion, but rather a central nerve lesion seemed to be the only way in which it could be produced. He would regard neuritis as secondary rather than causal. J. Hutchinson[34] points out that there are three types which must be distinguished: (1) True Raynaud's disease, where there is a morbid susceptibility to cold, which produces paroxysmal closure of the arteries; gangrene may occur in the paroxysms. (2) Symmetrical gangrene of the extremities, acro-sphacelus, with nothing paroxysmal about it, and complete recovery of the circulation after a single attack. (3) Cases in which the skin passes into a condition of diffuse scleroderma. Here again is a tendency to paroxysms and gangrene, but these are merely complications of the scleroderma. In true Raynaud's disease there is no such change in the skin. The best remedy for the pure disease is the habitual use of very small doses of opium. Another type is described by Lévi[35] as purely hysterical. It can be ameliorated by hypnotism, but tends to recur. There is often a previous history of rheumatic fever; the onset is sudden, and often accompanied by urinary disturbances, such as polyuria. It is one of the vaso-motor phenomena of hysteria, and gangrene sometimes takes place. Some cases of ainhum are described by H. De Brun. A toe is gradually detached without pain, hæmorrhage, or sore, by a constriction at the digito-plantar fold. The fifth toe generally is the first to go, and others follow, there is diminished temperature in the limb, some swelling and lividity, some sensory disturbances and loss of knee jerk, and the paroxysmal character found in Raynaud's disease. It nearly always occurs in the coloured races. Angiopathic gangrenes must, according to Lancereaux, be carefully distinguished from angio-neurotic.[37] The former are marked by severe nerve pains for years beforehand, after which mortified patches or phlyctenæ occur, which never terminate in moist gangrene. Raynaud's disease he looks upon as a special form of gangrene due to some usually undetected form of intoxication. The influence of cold and other agencies in producing functional spasm is, he thinks, exaggerated.

17 Am. M.S. Bullet., Sept. 1. 18 Int. Med. Mag., July. 19 Fortsch. der Med., p. 554, 1894. 20 A. J. Med. Sc., Nov. 21 Birming. Med. Rev., Sept. 1. 22 Lancet, Sept. 29. 23 Lancet, Oct. 6. 24 Lancet, Dec. 29. 25 Lancet, Nov. 24. 26 B.M.J., July 7. 27 Lancet, Jan. 5, 1895. 28 B.M.J., July 7. 29 B.M.J., Aug. 17. 30 Lancet, Jan. 5, 1895. 31 B.M.J., Oct. 20. 32 Int. Med. Mag., July. 33 Lancet, Jan. 5, 1895. 34 Clinica, J., Nov. 28. 35 Lancet, Dec. 1. 36 Med. Week, Sept. 28. 37 Med. Weekl June 25.

